# Gastroesophageal Reflux Disease-Associated Chronic Cough: A Population-Based Analysis of Patient Presentations in the United States

**DOI:** 10.7759/cureus.17512

**Published:** 2021-08-27

**Authors:** Fahad Qureshi, Hussein Asad, Parth S Patel, Aarya Ramprasad, Som P Singh, Sahil Suman, An-Lin Cheng, Gary Salzman

**Affiliations:** 1 Medicine, University of Missouri–Kansas City School of Medicine, Kansas City, USA; 2 Pulmonary and Critical Care, University of Missouri–Kansas City, Kansas City, USA; 3 Internal Medicine, University of Missouri–Kansas City School of Medicine, Kansas City, USA; 4 Epidemiology and Public Health, University of Missouri–Kansas City School of Medicine, Kansas CIty, USA; 5 Bioinformatics, University of Missouri–Kansas City, Kansas City, USA; 6 Biomedical and Health Informatics, University of Missouri–Kansas City School of Medicine, Kansas City, USA; 7 Internal Medicine, University of Missouri–Kansas City/Truman Medical Center, Kansas City, USA

**Keywords:** chronic cough, gastro-esophageal reflux disease, aspiration, proton pump inhibitor, reflux, asthma

## Abstract

Gastroesophageal reflux disease is an extremely prevalent illness in the United States; however, clinicians report that its association with chronic cough is often overlooked and undiagnosed. We used the CERNER Health Facts^®^ database to analyze the statistical prevalence. Our findings indicate that there is a minority of patients who are untreated for this common complaint. We propose considering this on the differential diagnosis and following current treatment guidelines with proton pump inhibitors to effectively treat this complaint.

## Introduction

Chronic cough is a common complaint responsible for approximately 30 million patient visits a year in the United States alone [1]. In the outpatient setting, evaluation of cough can account for up to 40% of volume [2]. The high prevalence of such cough may be in part from its variable etiologies. In the United States, the top four causes of chronic cough, in no particular order, are upper airway cough syndrome, asthma, non-asthmatic eosinophilic bronchitis, and gastroesophageal reflux disorder, which will henceforth be referred to as GERD [3].

The Montreal definition of GERD is as follows: “a condition which develops when the reflux of stomach content causes troublesome symptoms and/or complications” [4]. Although not an overt component of the pulmonary system, the pathophysiological role of GERD in chronic cough presentation has been linked to two predominant mechanisms: vagally mediated esophageal-tracheobronchial reflex (i.e., Reflex Theory) and micro-aspiration of gastric contents (i.e., Reflux Theory) [5]. In a study exploring vagally mediated esophageal-tracheobronchial reflex, the intra-esophageal acid infusion was shown to significantly increase sensitization to the cough reflex in patients suffering from chronic cough and asthma. However, it was noted that such hypersensitization occurred only in patients with overt evidence of GERD and airway disease, such as asthma, and not if patients presented without an airway disease [6]. Hence, as Kahrilas et al. noted, the evidence suggests that both pathologies are a prerequisite to a chronic cough presentation [7].

Micro-aspiration, or regurgitation, occurs when minuscule amounts of gastric acid, enzymes, or bile reach the larynx and pharynx, referred to as laryngopharyngeal reflux (LPR). LPR is a frequent cause of chronic laryngitis, which presents with a broad range of symptoms including chronic cough [7]. Although the micro-aspiration theory is debated, namely whether cough or reflux is the inciting factor for the other, the current hypothesis is that reflux-cough patients exhibit both airway and esophageal hypersensitivity [8]. 

The most classical symptom of GERD is heartburn, but extraesophageal symptoms resulting from the aforementioned mechanisms may also be present. Associated symptoms may include hoarseness, globus sensation, sour taste, and bronchospasm, leading to asthma or chronic cough [9].

Such patient presentations are unlikely to decrease anytime soon as common risk factors for GERD, including obesity, worsening diets, and decreased physical activity, among others, have drastically increased in the past few decades [10]. In the United States, the age-adjusted prevalence of obesity (BMI at or above 30.0) has increased from 30.5% in 1999-2000 to 42.4% in 2017-2018 [11]. These factors have resulted in a GERD prevalence range estimate of 18.1%-27.8% in North America as well as a larger portion of younger patients being diagnosed with GERD [12,13]. Hence, the role of GERD in chronic cough presentation may only increase in incidence and severity.

Nonetheless, despite previous studies indicating its role in chronic cough, a large-scale patient population-based study analyzing the epidemiological impact of GERD alone and in conjunction with other chronic cough etiologies is yet to be conducted in the United States.

## Materials and methods

As this study aimed to analyze the prevalence of GERD-mediated chronic cough through a number of factors, a retrospective analysis of patient data was utilized. In order to conduct such a retrospective analysis of a large patient population, the CERNER Health Facts^®^ database was employed, collecting both inpatient and outpatient encounter samples of patients presenting with chronic cough (inclusion criteria: patients of age ≥ 18 years presenting with a chief complaint of “cough”). Unique de-identified numbers were then assigned to each individual patient (different than patient medical record numbers), in lieu of each patient encounter, to alleviate the issue of counting a single individual more than once and to uphold patient privacy. In regard to stratifying single/multiple patient encounters, the following patient consolidation methodology was employed: Patients with multiple encounters were combined into a single patient record (i.e., data point) with multiple, non-overlapping values grouped together and overlapping values using the most recent information. For information absent in the database (e.g., BMI), the respective data was not included in the gross analysis. On the whole, a total of 457,105 different patient encounters were collected.

Patients then were categorized into sections based upon their chief complaint - referenced using ICD10 codes - to understand the varying causes and respective prevalence of the main causes of chronic cough. For this study, the chronic cough was defined as lasting more than three weeks and excluded upper respiratory infections, viral/bacterial infections, and acute lower respiratory infections (excluded etiologies are referred to as “Other”). Hence, for lower chronic cough, the following causes were analyzed: asthma (ICD10 code of J45), chronic obstructive pulmonary disease (COPD) (ICD10 codes of J40, J41, J42, J43, J44, J47), and GERD (ICD10 code of K21). As a note, the category COPD encompassed the following conditions: bronchitis, simple and mucopurulent chronic bronchitis, unspecified chronic bronchitis, emphysema, other chronic obstructive pulmonary disease, and bronchiectasis. Additionally, the gender breakdown and impact of obesity, measured through a BMI ≥ 30, were analyzed as well.

In order to further evaluate the methods of treating a chronic cough presentation, the medications prescribed to each patient were noted. As various forms of medication classes exist, the specific prescriptions were grouped under distinct categories as described in Table 1.

**Table 1:** Evidence-based pharmacological treatments of asthmatic versus gastroesophageal reflux cough GERD, Gastroesophageal reflux disease.

Asthmatic Cough			GERD Cough		
Beta-agonist bronchodilators	Corticosteroids	Anticholinergics	Antacids	Proton pump inhibitors	H2 blockers
Albuterol	Budesonide	Tiotropium	Calcium carbonate	Esomeprazole	Famotidine
Albuterol Ipratropium	Budesonide - Inhaled	Umeclidinium		Lansoprazole	Ranitidine
Levalbuterol	Budesonide - Nasal	Umeclidinium vilanterol		Omeprazole	
Budesonide formoterol - Inhaled	Budesonide formoterol - Inhaled			Pantoprazole	
Fluticasone falmeterol - Inhaled	Fluticasone - Inhaled			Rabeprazole	
Fluticasone vilanterol - Inhaled	Fluticasone - Nasal				
Formoterol	Fluticasone - Topical				
Formoterol mometasone	Fluticasone salmeterol - Inhaled				
Umeclidinium vilanterol	Fluticasone vilanterol - Inhaled				
	Formoterol mometasone				

For this study, as referred to in common practice, beta-agonists and corticosteroids may be used interchangeably for COPD or asthma; anticholinergics are typically used for COPD and may be used in severe uncontrolled asthma; antacids, proton pump inhibitors (PPIs), and H2 blockers are used for GERD alone in this study. A cross-tabulation analysis of patient medications was correspondingly conducted to evaluate if patients presenting with chronic cough were evaluated receiving medication.

Hence, for simplicity’s sake, the categories were named as follows (referred to in Table 4): “GERD Medication Alone” and “GERD Medication + Asthma/COPD Medication” with a small number of patients who were diagnosed with GERD but not given GERD-specific medications labeled “GERD Untreated.”

## Results

Due to the fact that the diagnosis of cough is challenging by itself and the fact that the differential diagnosis is vast, it was noted that >80% of causes for cough were labeled as “others.” However, among the identified etiologies, it was third to Asthma and COPD. The top three most commonly identified etiologies for cough were as follows: asthma at 8.8% of all cases (40,096/457,105), followed by COPD at 6.8% (30,960/457,105), and finally by GERD at 5.2% (23,804/457,105). This is shown in Table 2.

**Table 2:** Breakdown of cough etiologies (non-exclusive)

Disease	Number of Patients	Percentage of Patients
Asthma	40,096/457,105	8.8%
COPD	30,960/457,105	6.8%
GERD	23,804/457,105	5.2%

To identify patients with GERD and cough as presenting symptoms, we filtered results and queries to include only patients who had cough and GERD without asthma or COPD. The results were quite surprising in the fact that 5.2% (23,802/457,105) of patients had cough secondary to GERD. There was a statistically significant increased prevalence of GERD among females (5.5% vs. 4.7%), and as expected, the incidence of GERD was higher as BMI increased in 8.6% of patients with GERD having a BMI of ≥30. This is shown in Table 3.

**Table 3:** Breakdown of GERD-associated cough to gender GERD, Gastroesophageal reflux disease.

Gender	Number of Patients With GERD	Percentage of Patients With GERD
Female	15,580/282,272	5.5%
Male	8,222/174,762	4.7%
Total	23,802/457,105	5.2%

It was noted that 3.2% of patients with chronic cough were on GERD medications alone, and only 1.8% were on concomitant therapies for GERD and asthma/COPD. Approximately 0.2% of patients carried the diagnosis of GERD but had no therapies targeted to address that issue. This is shown in Table 4.

**Table 4:** Breakdown of chronic cough treatment (GERD ± exclusive) GERD, Gastroesophageal reflux disease; COPD, chronic obstructive pulmonary disease.

Disease Treatment	Number of Patients	Percentage of Patients
GERD medication alone	14,646/457,105	3.2%
GERD medication + asthma/COPD medication	8,250/457,105	1.8%
GERD untreated	906/457,105	0.2%

## Discussion

Chronic cough is a challenging disease entity that is incompletely understood. Despite all the advances in diagnostic testing available to the clinician, it continues to be a hassle. Well-known causes are obstructive airway diseases along with other pulmonary and non-pulmonary issues. However, GERD is a serious offender as it is one of the most common gastrointestinal disorders with a reported prevalence of approximately 20% in the United States [14]. Furthermore, it has been reported to be underestimated as access to over-the-counter anti-acid therapies has been increasing [12]. The fact that GERD is responsible for recurrent micro-aspiration linked to chronic cough and has been reported doing so in the literature [15], we should consider starting therapies with acid-reducing agents in patients with chronic cough.

Our study is an observational model of the United States that has shown a significant number of untreated chronic cough patients with GERD. The evidence for PPIs as a treatment of chronic cough is lacking; however, acid-reducing agents have been utilized and have been shown to improve chronic cough. Due to the safety of these medications and the limited side effects, we propose it is reasonable to consider GERD therapy a part of chronic cough therapy after careful screening and evaluation.

Regarding treatment for GERD, there is some contention on whether proton pump inhibitors effectively improve chronic cough presentation. Of note, antacid treatment, however, has shown to improve chronic cough.

Although several uncontrolled studies have shown improvement of chronic cough with antacid treatment, more recent randomized controlled trials have shown no differences between PPIs and placebo [16]. Although there is poor evidence that PPIs are universally beneficial for GERD-induced chronic cough, consensus guidelines recommend empiric therapy for at least eight weeks in conjunction with lifestyle changes such as dietary changes and weight loss [17]. The addition of a histamine H2 receptor antagonist and/or baclofen (Lioresal, 20 mg per day) may be helpful [18,19].

## Conclusions

Our findings revealed that there is a notable minority who is not being effectively treated for complications of GERD in the form of cough. Though the affected percentage may be small (0.2%), this number becomes significant when one realizes that up to 100 million Americans may feel the effects of reflux. In brief, though the relative number initially appears small, the absolute number is enormous. It is critical that this remains on the differential diagnosis especially in populations found to have an increased positive predictive value.

Finally, and perhaps of greatest significance, we propose that clinicians include GERD complications among the top three differentials for a persistent cough. Our findings indicate that of factors searchable in electronic medical records, GERD complications are the primary cause of non-pulmonary persistent cough. This is surprising because it has been historically overlooked, theorized to be due to the increase in Americans with GERD as obesity has become more prevalent. In summary, we hope that clinicians will consider GERD complications when presented with patients complaining of chronic cough in line with the statistical outcomes discussed above.

## References

[1] Irwin RS, Baumann MH, Bolser DC (2006). Diagnosis and management of cough executive summary: ACCP evidence-based clinical practice guidelines. Chest.

[2] Irwin RS, French CT, Lewis SZ, Diekemper RL, Gold PM (2014). Overview of the management of cough: CHEST Guideline and Expert Panel Report. Chest.

[3] Irwin RS, Curley FJ, French CL (1990). Chronic cough. The spectrum and frequency of causes, key components of the diagnostic evaluation, and outcome of specific therapy. Am Rev Respir Dis.

[4] Vakil N, van Zanten SV, Kahrilas P, Dent J, Jones R (2006). The Montreal definition and classification of gastroesophageal reflux disease: a global evidence-based consensus. Am J Gastroenterol.

[5] Naik RD, Vaezi MF (2015). Extra-esophageal gastroesophageal reflux disease and asthma: understanding this interplay. Expert Rev Gastroenterol Hepatol.

[6] Javorkova N, Varechova S, Pecova R (2008). Acidification of the oesophagus acutely increases the cough sensitivity in patients with gastro-oesophageal reflux and chronic cough. Neurogastroenterol Motil.

[7] Kahrilas PJ, Smith JA, Dicpinigaitis PV (2014). A causal relationship between cough and gastroesophageal reflux disease (GERD) has been established: a pro/con debate. Lung.

[8] Smith JA, Decalmer S, Kelsall A (2010). Acoustic cough-reflux associations in chronic cough: potential triggers and mechanisms. Gastroenterology.

[9] Clarrett DM, Hachem C (2018). Gastroesophageal reflux disease (GERD). Mo Med.

[10] El-Serag H (2008). The association between obesity and GERD: a review of the epidemiological evidence. Dig Dis Sci.

[11] Hales CM, Carroll MD, Fryar CD, Ogden CL (2020). Prevalence of obesity and severe obesity among adults: United States, 2017-2018. NCHS Data Brief.

[12] El-Serag HB, Sweet S, Winchester CC, Dent J (2014). Update on the epidemiology of gastro-oesophageal reflux disease: a systematic review. Gut.

[13] Yamasaki T, Hemond C, Eisa M, Ganocy S, Fass R (2018). The changing epidemiology of gastroesophageal reflux disease: are patients getting younger?. J Neurogastroenterol Motil.

[14] Antunes C, Aleem A, Curtis SA (2021). Gastroesophageal Reflux Disease. https://pubmed.ncbi.nlm.nih.gov/28722967/.

[15] Bonatti H, Achem SR, Hinder RA (2008). Impact of changing epidemiology of gastroesophageal reflux disease on its diagnosis and treatment. J Gastrointest Surg.

[16] Irwin RS, Zawacki JK, Curley FJ, French CL, Hoffman PJ (1989). Chronic cough as the sole presenting manifestation of gastroesophageal reflux. Am Rev Respir Dis.

[17] Chang AB, Lasserson TJ, Gaffney J, Connor FL, Garske LA (2011). Gastro-oesophageal reflux treatment for prolonged non-specific cough in children and adults. Cochrane Database Syst Rev.

[18] Xu X, Lv H, Yu L, Chen Q, Liang S, Qiu Z (2016). A stepwise protocol for the treatment of refractory gastroesophageal reflux-induced chronic cough. J Thorac Dis.

[19] Koufman JA (2002). Laryngopharyngeal reflux 2002: a new paradigm of airway disease. Ear Nose Throat J.

